# Genome wide analysis of novel copy number variations duplications/deletions of different epileptic patients in Saudi Arabia

**DOI:** 10.1186/1471-2164-16-S1-S10

**Published:** 2015-01-15

**Authors:** Muhammad Imran Naseer, Muhammad Faheem, Adeel G Chaudhary, Taha A Kumosani, Maha Mohsin Al-Quaiti, Mohammed M Jan, Hasan Saleh Jamal, Mohammad H Al-Qahtani

**Affiliations:** 1 Center of Excellence in Genomic Medicine Research, King Abdulaziz University, Jeddah, KSA; 2 KACST Technology Innovation Center in Personalized Medicine, King Abdulaziz University, Jeddah, Kingdom of Saudi Arabia; 3 Department of Biochemistry, Faculty of Science, King Abdulaziz University, Jeddah, KSA; 4 King Fahd Medical Research Center, King Abdulaziz University, Jeddah, KSA; 5 Department of Pediatrics, Faculty of Medicine, King Abdulaziz University, Box 80215, Jeddah 21589, Kingdom of Saudi Arabia; 6 Department of Obsttrics & Gynecology, King Abdulaziz University Jeddah

## Abstract

**Background:**

Epilepsy is genetically complex neurological disorder affecting millions of people of different age groups varying in its type and severity. Copy number variants (CNVs) are key players in the genetic etiology of numerous neurodevelopmental disorders and prior findings also revealed that chromosomal aberrations are more susceptible against the pathogenesis of epilepsy. Novel technologies, such as array comparative genomic hybridization (array-CGH), may help to uncover the pathogenic CNVs in patients with epilepsy.

**Results:**

This study was carried out by high density whole genome array-CGH analysis with blood DNA samples from a cohort of 22 epilepsy patients to search for CNVs associated with epilepsy. Pathogenic rearrangements which include 6p12.1 microduplications in 5 patients covering a total region of 99.9kb and 7q32.3 microdeletions in 3 patients covering a total region of 63.9kb were detected. Two genes *BMP5* and *PODXL* were located in the predicted duplicated and deleted regions respectively. Furthermore, these CNV findings were confirmed by qPCR.

**Conclusion:**

We have described, for the first time, several novel CNVs/genes implicated in epilepsy in the Saudi population. These findings enable us to better describe the genetic variations in epilepsy, and could provide a foundation for understanding the critical regions of the genome which might be involved in the development of epilepsy.

## Background

Epilepsy is one of the most common neurological disorder in humans with 1% prevalence and a lifetime incidence of up to 3%; characterized by recurrent and unprovoked seizures because of an abnormal electrical activity in central nervous system (CNS) [1]. More than 50 distinct epilepsy syndromes have been recognized with a broad range of clinical features; roughly it can be divided into idiopathic or symptomatic epilepsies. Metabolic disorders, infections, stroke, head trauma or brain tumors may cause symptomatic epilepsy whereas idiopathic seizure occur mainly because of genetic contribution [2]. Since long, it is observed that idiopathic epilepsy has a genetic component and its genetic etiology can be determined in a small fraction of cases. Development of genome wide technologies such as array-CGH and single-nucleotide polymorphism genotyping enabled the detection of submicroscopic microdeletions and microduplications also known as CNVs [3,4]. CNVs being an important component of human genetic variation are key players in the genetic etiology of numerous neurodevelopmental disorders. A number of studies have recently highlighted the role of CNVs in the etiology of various disorders including autism [5,6], intellectual disability (ID) [7] and schizophrenia [8,9]. Recent studies on six genomic regions showed recurrent microdeletions on chromosomes 15q13.3, 16p13.11 and 15q11.2 and were identified as necessary genetic factors influencing idiopathic generalized epilepsy (IGE) [10-12]. Databases of normal and pathogenic genome variations are available on the web and are extremely valuable tools for interpreting CNVs identified in patients (Database of Genomic Variants: DGV; Database of Chromosomal Imbalance and Phenotype in Humans Using Ensembl Resources: DECIPHER). Recent studies showed that 267 different genomic loci have been associated with well described microdeletion/microduplication syndromes (MMSs) [13]; these informations are constantly updated. In this study, we hypothesized that epilepsy could be caused by CNVs and that genes within those CNVs would be novel candidate genes for epilepsy. We selected a cohort of 22 patients with different types of epilepsies and performed high density whole genome array-CGH which showed novel CNVs/genes deletion and duplication that might be the contributory factors in the genetic etiology of epilepsy.

## Results and discussion

Array-CGH results showed gains as well as losses in different genomic regions of 19 epilepsy patients from a cohort of 22 epilepsy patients. But, we reported the results of only 8 patients satisfying the cut off value of duplications and deletions (0.8 for duplication and -1.0 for deletion). Microduplication of 6p12.1 was observed in five patients including two from the same family and microdeletion of 7q32.3 in three patients was also found. These CNV findings were confirmed by qPCR.

### Detection of 6p12.1 microduplications

**Figure 1:**
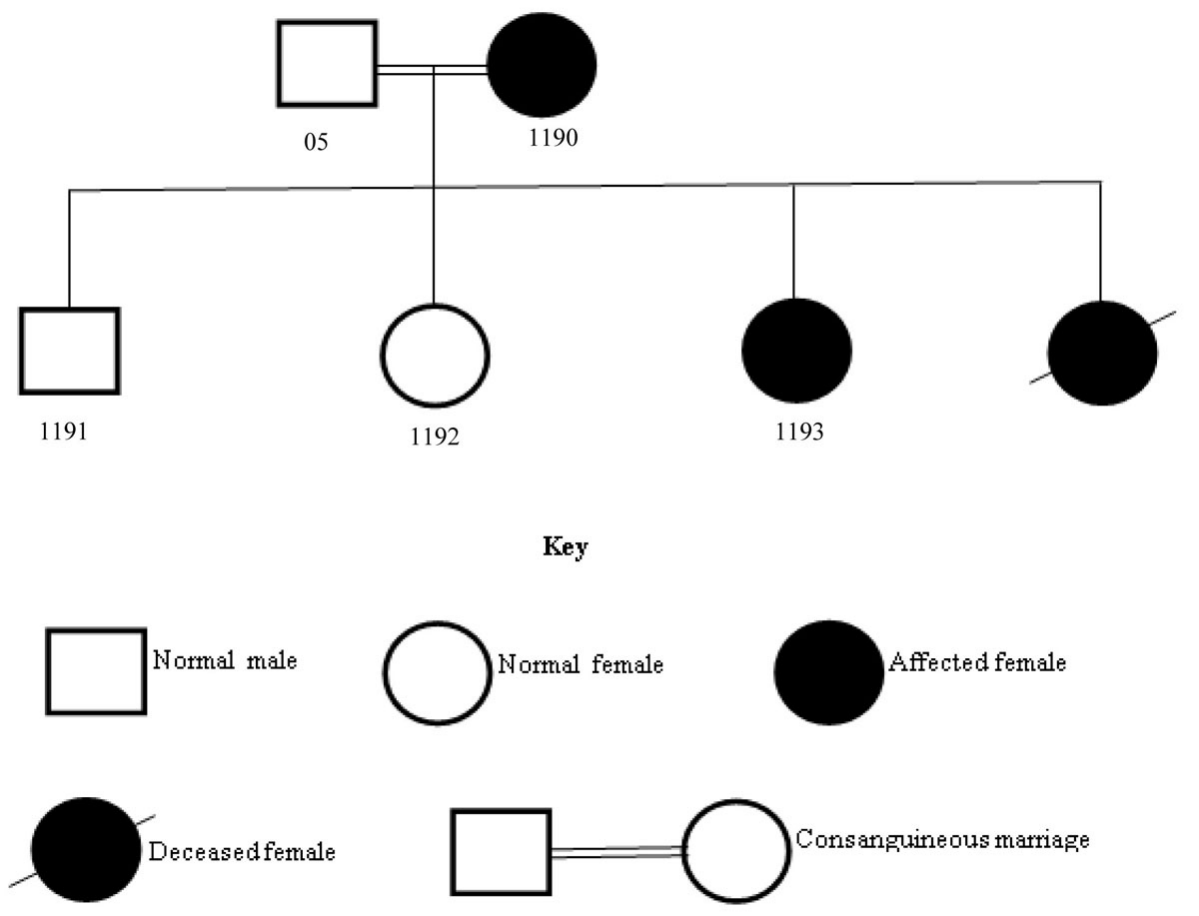
Pedigree of the epilepsy family; 05: normal father; 1190: affected mother; 1191: normal son: 1192: normal daughter: 1193: affected daughter.

**Figure 2:**
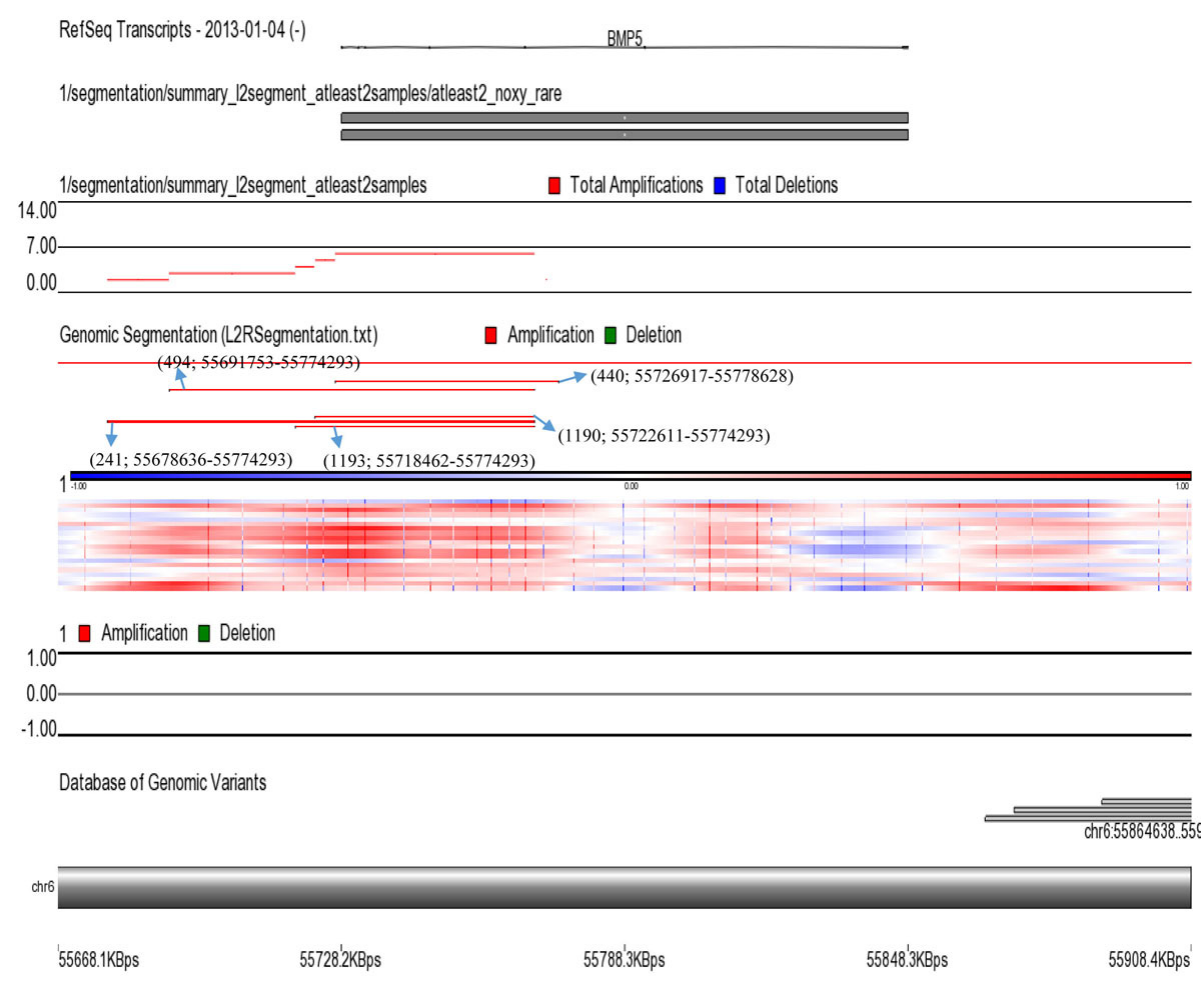
Summary of genome wide gains in five patients. Whole genome 2x 400K oligonucleotide based microarray analysis showing 99.9kb duplication at cytoband 6p12.1. Five horizontal red lines showing duplicated regions of five patients; each red line is marked with each patients ID along with the duplicated region: 1193; 55718462-55774293, 241; 55678636-55774293, 1190; 55722611-55774293, 494; 55691753-55774293 and 440; 55726917-55778628. The gene predicted in the duplicated region is *BMP5.*

**Figure 3:**
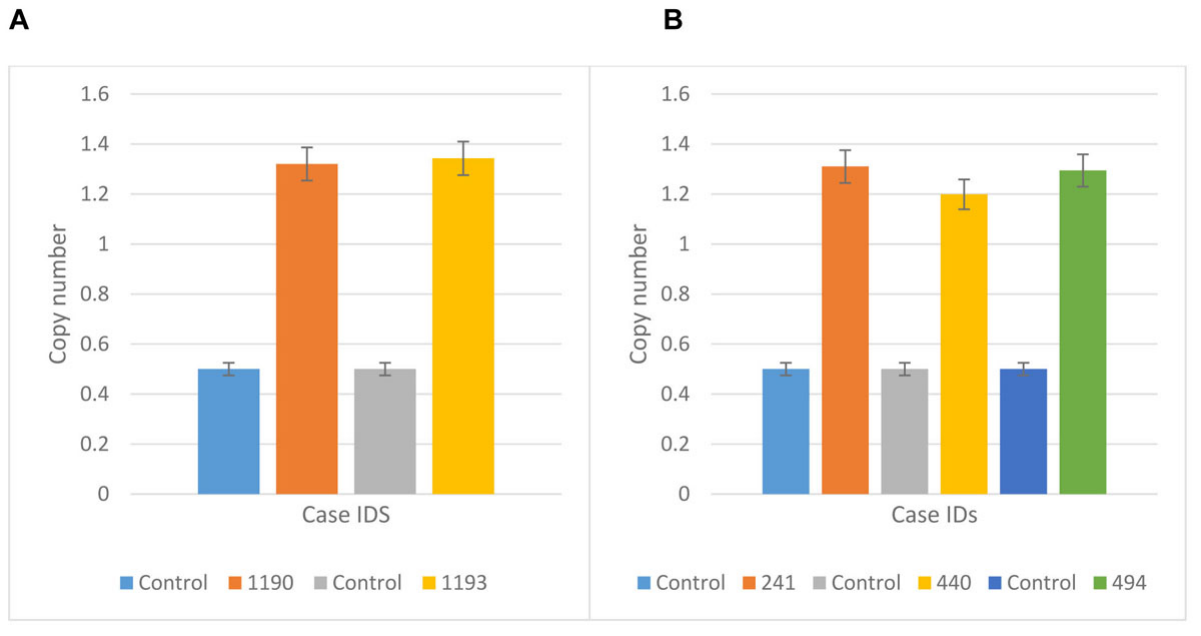
Confirmation of CNVs by qPCR. (A) The results showed *BMP5* copy number amplifications in two patients; mother (1190) and the proband (1193) but not in healthy individuals. (B) *BMP5* copy number amplifications were observed in three patients (241, 440 and 494) but not in healthy individuals.

Whole genome 2x 400K oligonucleotide based microarray analysis showed 99.9kb duplication at cytoband 6p12.1. Figure 2 presented 5 horizontal red lines of 5 patients with duplicated regions; each red line is marked with each patients ID along with the duplicated region of that specific patient. Out of these 5 patients, 2 were members of the same family whereas the rest of 3 were sporadic cases. No deletion or duplication was observed in father: 05, normal daughter: 1192 and normal son: 1191 (Figure 1). In affected mother: 1190, a microduplication of 51.6kb (55722611-55774293) and in affected daughter: 1193, a microduplication of 55.8kb (55718462-55774293) was observed. Rest of 3 sporadic cases also showed duplications of different sizes such as 241 showed a duplication of 95.6kb (55678636-55774293); 494 showed a duplication of 82.5kb (55691753-55774293) and 440 showed a duplication of 51.7kb (55726917-55778628). The gene that was observed in the duplicated region of all the patients is *BMP5* (Table 1 and Figure 2); determined by UCSC Genome Browser (http://genome.ucsc.edu/) and Database of Genomic Variants (http://dgv.tcag.ca/dgv/app/home). *BMP5* is a member of BMP family which belongs to transforming growth factor-β (TGFβ) superfamily. It is expressed in hippocampus, cerebellum and striatum [14,15]. Studies also showed its expression in developing rat superior cervical ganglion corresponding to initial extension of primary dendrites and in early postnatal period during maximal dendritic growth. Immunofluorescence analysis of cultured superior cervical ganglion cells detected *Bmp5* all over cell body of glial [16]. It was also observed that cell proliferation was reduced by *BMP5* through cell death into the progenitor cells specifically in ventral forebrain. Insertion of *BMP5* in developing chicken prosencephalon causes loss of ventral forebrain secondary to immense cell death localized to that region. Experimental embryos showed a loss of basal telencephalon due to implantation of beads soaked in recombinant *BMP5* or *BMP4* into neural tube of chicken forebrain that resulted in holoprosencephaly (a single cerebral hemisphere), cyclopia (a single midline eye) and loss of ventral midline structures [17]. Noradrenergic (NA) neurons are critical modulators of brain functions and have been implicated in common CNS disorders [18]. Locus coeruleus (LC), the major NA nucleus providing the main source of noradrenaline in the brain is formed in rostral hindbrain during embryogenesis. LC development requires either *Bmp5* or *Bmp7* and one is able to compensate the loss of the other. Also, the position of mid-hindbrain organizer determines the size of LC and propose that *Bmp5/7* play an important role in mediating this organizer function [19]. Studies also uncovered novel roles of BMP signaling during development of heart, allantois, branchial arches, somites and forebrain development. *Bmp5* do not appear to be involved in establishing pattern in these tissues but necessary for proliferation and maintenance of specific cell populations [20]. Observed duplications in all patients with reference to controls were confirmed by qPCR which showed a significant fold increase in all patients compared to controls Figure 3).

**Table 1:** Characteristics of study participants carrying copy number variations (duplications and deletions).

Case No	Sex	Location	CNVs		Change	Genes	IGE Syndrome
			Start (bp)-Stop (bp)	Size (kb)			
1193	F	6p12.1	55718462-55774293	55.8	Duplication	*BMP5*	Epi
241	M	6p12.1	55678636-55774293	95.6	Duplication	*BMP5*	Epi
1190	F	6p12.1	55722611-55774293	51.6	Duplication	*BMP5*	Epi
494	M	6p12.1	55691753-55774293	82.5	Duplication	*BMP5*	Epi+MR
440	F	6p12.1	55726917-55778628	51.7	Duplication	*BMP5*	JME
1090	F	7q32.3	130860344-130924315	63.9	Deletion	*PODXL*	Epi
1186	M	7q32.3	130864738-130924315	59.5	Deletion	*PODXL*	JME
495	F	7q32.3	130864738-130921416	56.6	Deletion	*PODXL*	Epi+MR

**Table legend text:** M: Male; F: Female; CNVs: Copy number variations; bp: base pairs; kb: kilobases; *BMP5*: Bone morphogenetic protein-5; *PODXL:* Podocalyxin-like protein; IGE: Idiopathic generalized epilepsy; Epi: Epilepsy; JME: Juvenile myoclonic epilepsy; Epi+MR: Epilepsy plus mental retardation.

### Detection of 7q32.3 microdeletions

**Figure 4:**
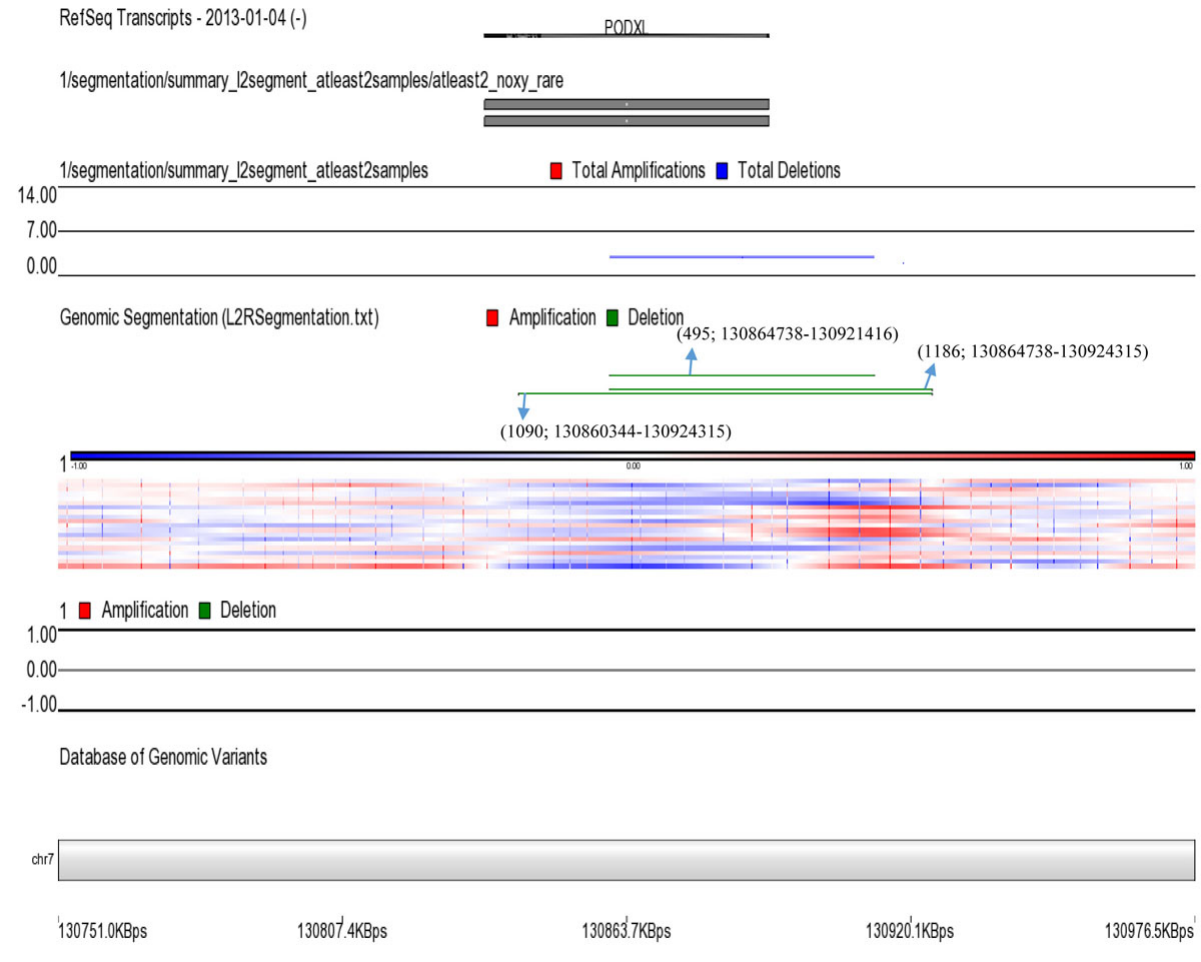
Summary of genome wide losses in three patients. Whole genome 2x 400K oligonucleotide based microarray analysis showing 63.9kb deletion at cytoband 7q32.3. Three horizontal green lines showing deleted regions of three patients; each green line is marked with each patients ID along with the deleted region: 1090; 130860344-130924315, 1186; 130864738-130924315 and 495; 130864738-130921416. The gene which is observed in the deleted region is *PODXL.*

**Figure 5:**
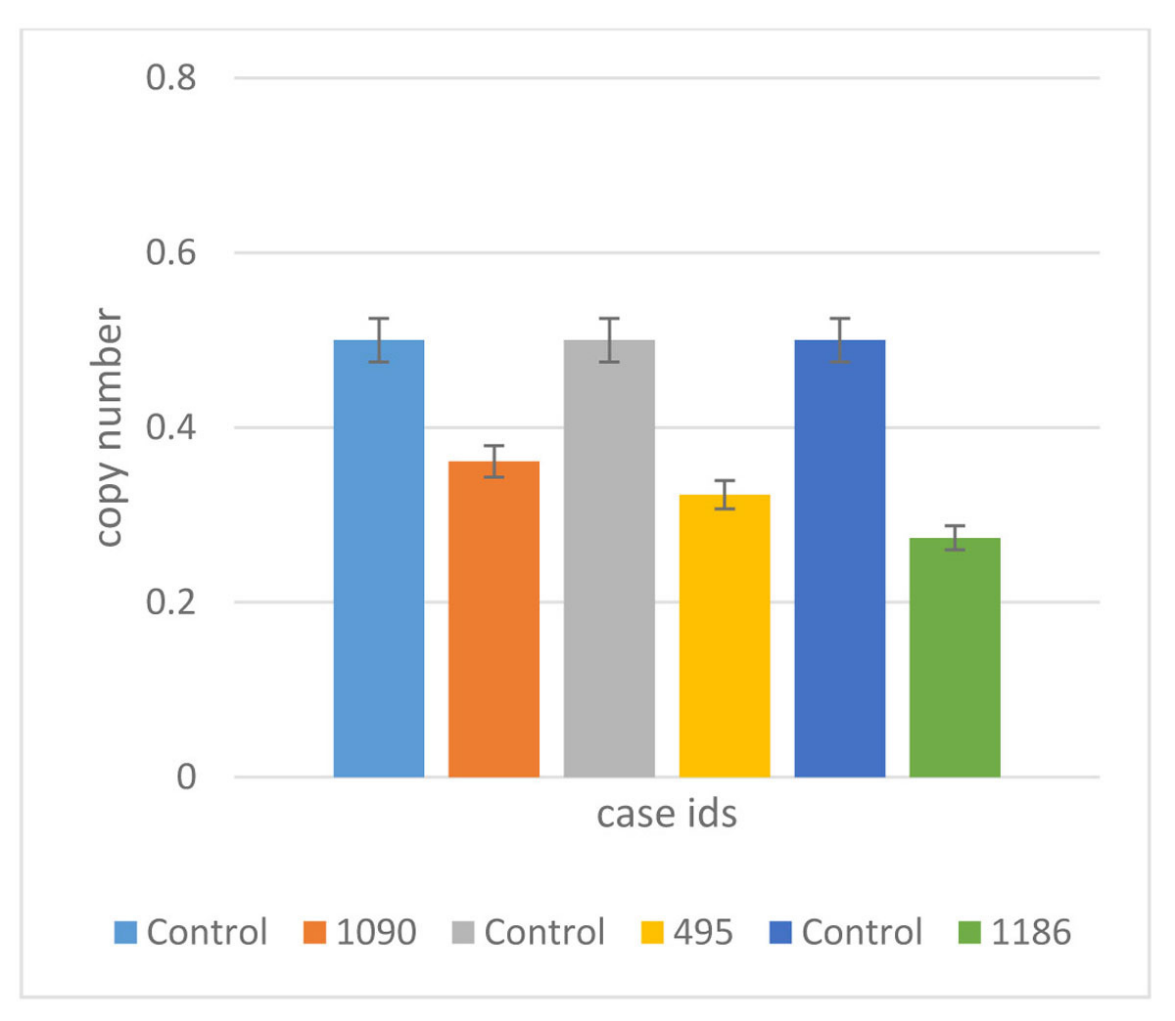
Confirmation of CNVs by qPCR. The results showed *PODXL* copy number deletions in three patients (1090, 495 and 1186) but not in healthy individuals.

Whole genome 2x 400K oligonucleotide based microarray analysis showed 63.9kb microdeletion at cytoband 7q32.3. Figure 4 showed 3 horizontal green lines of 3 patients with deleted regions; each green line is marked with each patients ID along with the deleted region of that specific patient. In patient 1090, a microdeletion of 63.9kb (130860344-130924315); in 1186, a microdeletion of 59.5kb (130864738-130924315) and in 495, a deletion of 56.6kb (130864738-130921416) was observed. The gene that was observed in the deleted regions of all the patients was *PODXL* (Table 1 and Figure 4); determined by UCSC Genome Browser (http://genome.ucsc.edu/) and Database of Genomic Variants (http://dgv.tcag.ca/dgv/app/home). Podocalyxin (*PODXL*) is type-1 membrane mucin protein belongs to CD34 family expressed abundantly in kidney epithelial cells (podocytes) [21]; its expression has also reported in the brain with maximum level in cortical plate, hippocampus, cerebellum and basal forebrain nuclei [22,23]. In humans, it is also linked with malignant progression of brain astrocytic tumors [24]. Studies also showed that ventricles of *Podxl^−/−^* mice were enlarged in coronal and horizontal brain MR images which were also confirmed through histological analysis of paraffin embedded brain [25]. Reason of this could be either an enhanced formation or reduced disposal of cerebrospinal fluid (CSF) or it might be due to a combination of both mechanisms. Enhanced volume of CSF might be the result of a new steady state equilibrium of choroid plexuses ion membrane transporters; *Na*^+^*-K*^+^*ATPase*, *K*^+^ channels and *Na*^+^*-2Cl^−^-K*^+^ co-transporters expressed in apical membrane and *Cl^−^-HCO3* exchangers, a variety of *Na*^+^ coupled *HCO3^−^* transporters and *K*^+^*-Cl^−^* cotransporters expressed in the basolateral membrane [26]. Further analysis is required to check the involvement of these ion transporters toward ventricles enlargement in the absence of *PODXL*. Numerous human pathological disorders are linked with enlarged ventricles such as trauma, autism, bardet-biedl syndrome and alzheimer disease [27-29]. However, an earlier and possibly best human pathological condition studied was schizophrenia [30]. Finding of enlarged ventricles in the patients having first schizophrenia episode has raised the question of whether this neurodegenerative disease exhibited at the time of symptom onset or it is considered as a neurodevelopmental process which produces abnormal brain volumes at an early age [31]. Observed deletions in all the patients with reference to controls were confirmed by qPCR which showed a significant fold decrease in all the patients compared to controls (Figure 5).

## Conclusion

CNVs are major contributory factors in developing the neurological disorders and also contribute in the genetic etiology of epilepsy. In this study, we described, for the first time, some of the novel CNVs/genes implicated in epilepsy in the Saudi population. Our findings present a better description of genetic variations in epilepsy, and could provide a foundation for the understanding of the critical regions of the genome which might be involved in the development of epilepsy. More comprehensive descriptions of patients and identification of possible causative CNVs and genes in the CNV regions will be required to identify new syndromes and improve the diagnoses of epilepsy.

## Methods

### Patients and families

22 patients were selected with primary diagnosis of different types of epilepsies including epilepsy (Epi), epilepsy plus mental retardation (Epi+MR) and juvenile myoclonic epilepsy (JME) shown in the Table 1. Details of all patients are as follows; Case 1190 and 1193: 1193 is a 4 years old female proband; her seizures were started at the age of 3 years. Her 33 years old mother 1190 had a consanguineous marriage and also suffering with seizures that were started at the age of 17 years. 05: father; 1192: sister and 1191: brother of the proband were normal and no CNV was found in any of these normal members of the family. One patient of the same family was died at the age of 5 years due to seizures (Figure 1). Case 440: She is 39 years old female patient of JME and her seizures were started the age of 16 years. Case 241: He is 14 years old male patient of JME and his seizures were started the age of 15 years. Case 1090: she is 15 years old female patient of epilepsy, her parents had a consanguineous marriage and both are normal. Blood samples of her parents, one normal sister and one normal brother was not available. Her seizures were started when she was 8 years old. Case 1186: He is 27 years old male patient of JME and his seizures were started the age of 16 years. Case 495: She is 13 years old female patient of epilepsy plus mental retarded. Her seizures were started at the age of 6 years. Case 494: He is 7 years old male patient of epilepsy plus mental retarded. His seizures were started the age of 3 years.

### DNA preparation

Blood samples from all the patients were taken at King Abdulaziz University Hospital with their informed consent after the approval of ethical committee. DNA was extracted by using QIAamp DNA blood mini kit (http://www.Qiagen.com) and was quantified by NanoDrop ND-10000 Spectrophotometer.

### Array-CGH analysis

Array-CGH analysis was done by Agilent sure print G3 Hmn CGH 2x 400K arrays (Agilent Technologies, Santa Clara, USA) following manufacturer protocol. Briefly, 500ng patient’s DNA and also reference DNA of the same sex (Promega, Madison, USA) were digested with *RsaI* (Promega, Madison, USA) and *AluI* (Promega, Madison, USA) for 2hrs at 37ºC. Furthermore, these samples were labeled by random primers through Agilent labeling kit (Agilent Technologies, Santa Clara, USA) following manufacturer guidelines. Patient’s DNA was labeled with Cy5-dUTP whereas reference DNA was labelled with Cy3-dUTP. Labeled products were purified by Microcon YM-30 filter units (Millipore, Billerica, USA). Patient and reference DNAs were mixed with Cot-1 DNA (Invitrogen, USA) blocking agent and hybridization buffer following by Agilent instructions. After denaturation at 95ºC and pre-annealing at 37ºC, hybridization was done at 65ºC for 40hrs. After the two washing steps, array was analyzed through Agilent Scanner (G2505C) and Feature Extraction Software (V.1.5.1.0). Data was analyzed by using Partek Genomics Suite Software, Cytogenomics Software (V.2.0.6.0; Agilent Technologies, USA) and publicly available DGV.

### Validation of CNVs by real-time quantitative PCR

The microdeletions and microduplications were confirmed by real-time quantitative PCR (qPCR). Targeted sequences were chosen for qPCR by using Primer-3 Software (V.0.4.0). Two pair of primers were selected for each of genomic region of two target genes; bone morphogenetic protein-5 (*BMP5*) and podocalyxin-like protein (*PODXL*); an endogenous gene β_2_-microglobulin (*B2M*) and control samples. PCR was done in 10μl reaction volume comprising of 05μl SYBR-Green qPCR master mix (KAPA Biosystems, USA), 10 pmol forward and reverse primers and 20ng genomic DNA. Reaction cycling conditions were as follows; denaturation at 95°C for 10minutes, annealing at 60°C for 1minute, holding stage at 60°C for 30seconds followed by 45 cycles at 60°C for 15seconds. Each run of 96 well plate included control samples, target genes, reference gene and non-template control for each gene. Plate was analyzed with StepOnePlus™ Real-Time PCR Systems (StepOnePlus™ Real-Time PCR, Applied Biosystems, Canada) raw data was acquired using Light Cycler 480 Software. Ct is the threshold cycle defined as the mean cycle at which the fluorescence curve reaches an arbitrary threshold; ΔCt is the difference between the Ct of the target gene and that of reference gene; ΔΔCt is ΔCt value of patients obtained by dividing the patient ΔCt value with ΔCt value of control sample. *T-test* with significant *P-*value <0.05 was used to find out statistical significance of predicted copy number alterations.

## Abbreviations

Array-CGH: Array comparative genomic hybridization; CNVs: Copy number variations; qPCR: Real-time quantitative PCR; CNS: Central nervous system; BMP5: Bone morphogenetic protein-5; PODXL: Podocalyxin-like protein; B2M: β_2_-microglobulin; IGE: Idiopathic generalized epilepsy; JME: Juvenile myoclonic epilepsy; Epi+MR: Epilepsy plus mental retardation; DGV: Database of Genomic Variant.

## Competing interests

The authors declare that they have no conflicts of interest.

## Authors' contributions

MIN, MF and AGC designed the study. MF and MIN performed the experiments, analyzed the data and wrote the manuscript, MMA performed microarray. AGC, TAK, MMJ, MHQ, HSJ contributed in writing and editing the manuscript. All authors read and approved the final manuscript.

## References

[B1] Hauser WA Annegers JF Rocca WA Descriptive epidemiology of epilepsy: contributions of population-based studies from Rochester, Minnesota Mayo Clinic proceedings 1996 71 6 576 586 10.4065/71.6.576 8642887

[B2] Davidsson J Collin A Olsson ME Lundgren J Soller M Deletion of the SCN gene cluster on 2q24.4 is associated with severe epilepsy: an array-based genotype-phenotype correlation and a comprehensive review of previously published cases Epilepsy research 2008 81 1 69 79 10.1016/j.eplepsyres.2008.04.018 18539002

[B3] Koolen DA Pfundt R de Leeuw N Hehir-Kwa JY Nillesen WM Neefs I Scheltinga I Sistermans E Smeets D Brunner HG Genomic microarrays in mental retardation: a practical workflow for diagnostic applications Human mutation 2009 30 3 283 292 10.1002/humu.20883 19085936

[B4] Gurnett CA Hedera P New ideas in epilepsy genetics: novel epilepsy genes, copy number alterations, and gene regulation Archives of neurology 2007 64 3 324 328 10.1001/archneur.64.3.324 17353374

[B5] Christian SL Brune CW Sudi J Kumar RA Liu S Karamohamed S Badner JA Matsui S Conroy J McQuaid D Novel submicroscopic chromosomal abnormalities detected in autism spectrum disorder Biological psychiatry 2008 63 12 1111 1117 10.1016/j.biopsych.2008.01.009 18374305 PMC2440346

[B6] Marshall CR Noor A Vincent JB Lionel AC Feuk L Skaug J Shago M Moessner R Pinto D Ren Y Structural variation of chromosomes in autism spectrum disorder American journal of human genetics 2008 82 2 477 488 10.1016/j.ajhg.2007.12.009 18252227 PMC2426913

[B7] Friedman JM Baross A Delaney AD Ally A Arbour L Armstrong L Asano J Bailey DK Barber S Birch P Oligonucleotide microarray analysis of genomic imbalance in children with mental retardation American journal of human genetics 2006 79 3 500 513 10.1086/507471 16909388 PMC1559542

[B8] Stefansson H Rujescu D Cichon S Pietilainen OP Ingason A Steinberg S Fossdal R Sigurdsson E Sigmundsson T Buizer-Voskamp JE Large recurrent microdeletions associated with schizophrenia Nature 2008 455 7210 232 236 10.1038/nature07229 18668039 PMC2687075

[B9] Walsh T McClellan JM McCarthy SE Addington AM Pierce SB Cooper GM Nord AS Kusenda M Malhotra D Bhandari A Rare structural variants disrupt multiple genes in neurodevelopmental pathways in schizophrenia Science 2008 320 5875 539 543 10.1126/science.1155174 18369103

[B10] de Kovel CG Trucks H Helbig I Mefford HC Baker C Leu C Kluck C Muhle H von Spiczak S Ostertag P Recurrent microdeletions at 15q11.2 and 16p13.11 predispose to idiopathic generalized epilepsies Brain : a journal of neurology 2010 133 Pt 1 23 32 19843651 10.1093/brain/awp262 PMC2801323

[B11] Dibbens LM Mullen S Helbig I Mefford HC Bayly MA Bellows S Leu C Trucks H Obermeier T Wittig M Familial and sporadic 15q13.3 microdeletions in idiopathic generalized epilepsy: precedent for disorders with complex inheritance Human molecular genetics 2009 18 19 3626 3631 10.1093/hmg/ddp311 19592580 PMC3465696

[B12] Helbig I Mefford HC Sharp AJ Guipponi M Fichera M Franke A Muhle H de Kovel C Baker C von Spiczak S 15q13.3 microdeletions increase risk of idiopathic generalized epilepsy Nature genetics 2009 41 2 160 162 10.1038/ng.292 19136953 PMC3026630

[B13] Weise A Mrasek K Klein E Mulatinho M Llerena JC Jr. Hardekopf D Pekova S Bhatt S Kosyakova N Liehr T Microdeletion and microduplication syndromes The journal of histochemistry and cytochemistry : official journal of the Histochemistry Society 2012 60 5 346 358 10.1369/0022155412440001 22396478 PMC3351230

[B14] Charytoniuk DA Traiffort E Pinard E Issertial O Seylaz J Ruat M Distribution of bone morphogenetic protein and bone morphogenetic protein receptor transcripts in the rodent nervous system and up-regulation of bone morphogenetic protein receptor type II in hippocampal dentate gyrus in a rat model of global cerebral ischemia Neuroscience 2000 100 1 33 43 10.1016/S0306-4522(00)00246-3 10996456

[B15] Harvey BK Hoffer BJ Wang Y Stroke and TGF-beta proteins: glial cell line-derived neurotrophic factor and bone morphogenetic protein Pharmacology & therapeutics 2005 105 2 113 125 10.1016/j.pharmthera.2004.09.003 15670622

[B16] Beck HN Drahushuk K Jacoby DB Higgins D Lein PJ Bone morphogenetic protein-5 (BMP-5) promotes dendritic growth in cultured sympathetic neurons BMC neuroscience 2001 2 12 10.1186/1471-2202-2-12 11580864 PMC56999

[B17] Golden JA Bracilovic A McFadden KA Beesley JS Rubenstein JL Grinspan JB Ectopic bone morphogenetic proteins 5 and 4 in the chicken forebrain lead to cyclopia and holoprosencephaly Proceedings of the National Academy of Sciences of the United States of America 1999 96 5 2439 2444 10.1073/pnas.96.5.2439 10051661 PMC26803

[B18] Berridge CW Waterhouse BD The locus coeruleus-noradrenergic system: modulation of behavioral state and state-dependent cognitive processes Brain research Brain research reviews 2003 42 1 33 84 10.1016/S0165-0173(03)00143-7 12668290

[B19] Tilleman H Hakim V Novikov O Liser K Nashelsky L Di Salvio M Krauthammer M Scheffner O Maor I Mayseless O Bmp5/7 in concert with the mid-hindbrain organizer control development of noradrenergic locus coeruleus neurons Molecular and cellular neurosciences 2010 45 1 1 11 10.1016/j.mcn.2010.05.003 20493948

[B20] Solloway MJ Robertson EJ Early embryonic lethality in Bmp5;Bmp7 double mutant mice suggests functional redundancy within the 60A subgroup Development 1999 126 8 1753 1768 10079236 10.1242/dev.126.8.1753

[B21] Kerjaschki D Sharkey DJ Farquhar MG Identification and characterization of podocalyxin--the major sialoprotein of the renal glomerular epithelial cell The Journal of cell biology 1984 98 4 1591 1596 10.1083/jcb.98.4.1591 6371025 PMC2113206

[B22] Vitureira N McNagny K Soriano E Burgaya F Pattern of expression of the podocalyxin gene in the mouse brain during development Gene expression patterns : GEP 2005 5 3 349 354 10.1016/j.modgep.2004.10.002 15661640

[B23] Lin WL Pang VF Liu CH Chen JY Shen KF Lin YY Yu CY Hsu YH Jou TS Pleomorphic extra-renal manifestation of the glomerular podocyte marker podocalyxin in tissues of normal beagle dogs Histochemistry and cell biology 2007 127 4 399 414 10.1007/s00418-006-0252-8 17180683

[B24] Hayatsu N Kaneko MK Mishima K Nishikawa R Matsutani M Price JE Kato Y Podocalyxin expression in malignant astrocytic tumors Biochemical and biophysical research communications 2008 374 2 394 398 10.1016/j.bbrc.2008.07.049 18639524

[B25] Nowakowski A Alonso-Martin S Gonzalez-Manchon C Larrucea S Fernandez D Vilar M Cerdan S Ayuso MS Parrilla R Ventricular enlargement associated with the panneural ablation of the podocalyxin gene Molecular and cellular neurosciences 2010 43 1 90 97 10.1016/j.mcn.2009.09.011 19837166

[B26] Brown PD Davies SL Speake T Millar ID Molecular mechanisms of cerebrospinal fluid production Neuroscience 2004 129 4 957 970 15561411 10.1016/j.neuroscience.2004.07.003 PMC1890044

[B27] Hardan AY Minshew NJ Mallikarjuhn M Keshavan MS Brain volume in autism Journal of child neurology 2001 16 6 421 424 10.1177/088307380101600607 11417608

[B28] Davis RE Swiderski RE Rahmouni K Nishimura DY Mullins RF Agassandian K Philp AR Searby CC Andrews MP Thompson S A knockin mouse model of the Bardet-Biedl syndrome 1 M390R mutation has cilia defects, ventriculomegaly, retinopathy, and obesity Proceedings of the National Academy of Sciences of the United States of America 2007 104 49 19422 19427 10.1073/pnas.0708571104 18032602 PMC2148305

[B29] Nestor SM Rupsingh R Borrie M Smith M Accomazzi V Wells JL Fogarty J Bartha R Alzheimer's Disease Neuroimaging I Ventricular enlargement as a possible measure of Alzheimer's disease progression validated using the Alzheimer's disease neuroimaging initiative database Brain : a journal of neurology 2008 131 Pt 9 2443 2454 18669512 10.1093/brain/awn146 PMC2724905

[B30] Johnstone EC Crow TJ Frith CD Husband J Kreel L Cerebral ventricular size and cognitive impairment in chronic schizophrenia Lancet 1976 2 7992 924 926 62160 10.1016/s0140-6736(76)90890-4

[B31] Steen RG Mull C McClure R Hamer RM Lieberman JA Brain volume in first-episode schizophrenia: systematic review and meta-analysis of magnetic resonance imaging studies The British journal of psychiatry : the journal of mental science 2006 188 510 518 10.1192/bjp.188.6.510 16738340

